# A dynamic plasma membrane proteome analysis of alcohol-induced liver cirrhosis

**DOI:** 10.1186/1477-5956-10-39

**Published:** 2012-06-08

**Authors:** Xiaofang Jia, Lin Yin, Yanling Feng, Xia Peng, Fang Ma, Yamin Yao, Xiaoqian Liu, Zhiyong Zhang, Zhenghong Yuan, Lijun Zhang

**Affiliations:** 1 Shanghai Public Health Clinical Center affiliated to Fudan University, Shanghai 201508, China; 2 Institute of Clinical Pharmacology, Pharmacogenetics Research Institute, Changsha, Hunan 410078, China

**Keywords:** Alcohol, Liver cirrhosis, Plasma membrane, Proteomics

## Abstract

Alcohol-induced injury has become one of the major causes for liver cirrhosis. However, the molecular mechanisms of ethanol-induced injury are not fully understood. To this end, we performed a dynamic plasma membrane proteomic research on rat model. A rat model from hepatitis to liver cirrhosis was developed. Plasma membrane from liver tissue with liver fibrosis stage of 2 and 4 (S2 and S4) was purified by sucrose density gradient centrifugation. Its purification was verified by western blotting. Proteins from plasma membrane were separated by two-dimensional electrophoresis (2DE) and differentially expressed proteins were identified by tandem mass spectrometry. 16 consistent differentially expressed proteins from S2 to S4 were identified by mass spectrometry. The expression of differentially expressed proteins annexin A6 and annexin A3 were verified by western blotting, and annexin A3 was futher verified by immunohistochemistry. Our research suggests a possible mechanism by which ethanol alters protein expression to enhance the liver fibrosis progression. These differentially expressed proteins might be new drug targets for treating alcoholic liver cirrhosis.

## Introduction

About 90% of ethanol is oxidized in liver, thus liver is highly susceptible to alcohol-induced injury. Although the clinical manifestations of alcoholic liver disease are well described, little is known about the molecular basis for liver injury. As a consequence of ethanol administration, great changes take place in liver cells, including plasma membrane (PM), mitochondrion and nucleus. In plasma membrane, alcohol can increase the membranes permeability and induce membranes defects [1-3], and cause an increase in the amount of all phospholipids, in surface charge density as well as in lipid peroxidation products [4]. For mitochondrion, the membrane potential is lost, followed by the activation of caspase-9 and −3, and the apoptosis of cells [5]. In all, ethanol-induced liver damage is a complex process. For molecular mechanism study, it is necessary to simplify the experimental process, and use high throughput and sensitive methods.

Subcellular proteomics have met this requirement. As shown in several mitochondrial proteome works [6,7], a lot of mitochondrial proteins including oxoglutarate dehydrogenase (lipoamide), ketoacyl-CoA thiolase, etc. were regulated due to ethanol-dependent hepatotoxicity [6]. Similarly, a cytoplasm and membrane proteomic study based on 2DE also showed that alcohol induced hyperacetylation of multiple proteins in the cytosol and membrane during the development of liver injury. Plasma membrane proteins acts as ‘doorbells’ and ‘doorways’, and play crucial roles in intercellular communication, cellular development, cell migration and drug resistance [8-11]. Our previous studies have used proteome technology to examine the early effects of alcohol to liver disease [12]. However, further studies should be done to take a more in-depth look at the proteome change in alcohol-induced cirrhosis.

In this work, we used a proteomic study based on 2DE to examine the PM proteome changes from hepatitis to liver cirrhosis. Rat liver samples, with fibrosis stage 2 and 4, were collected after ethanol treating for 6 and 9 weeks respectively. Proteins were separated by 2DE, and the differentially expressed proteins were identified by mass spectrometry. Annexin A3 (ANXA3) and annexin A6 (ANNXA6) with altered abundance were selected for further verification by western blotting and immunohistochemistry. Further investigation of the function mechanism of ANXA3 and ANXA6 in liver cirrhosis may yield new clues to the molecular mechanism of alcohol-induced liver disease. Furthermore, it will be more helpful for understanding alcohol-induced liver disease through studying other differentially expressed identified in this work.

## Experimental procedures

### Animal treatment

60 eight-week-old male Sprague–Dawley rats (180–200 g) were purchased from Center of Laboratory Animals, Shanghai Public Health Center, Shanghai, P. R. China. Ethical approval was received from Shanghai Public Health Clinical Center. All of the animal studies followed the relevant national legislation and local guidelines, and were performed at the Center of Laboratory Animals. The animals were housed 4 per cage in an animal room (temperature: 23 ± 2°C, relative humidity: 55 ± 5°C, and 12 h-light and 12 h-dark cycle) with unlimited access to food and water. The animals were subjected to the experiment after acclimation for one week.

The rats were randomly divided into three groups including one for alcoholic group (24 rats) and one for control (24 rats), and the other for checking the function of pyrazole and olive oil (12 rats). The alcoholic group was intragastrically administrated with the complex containing sixty percent ethanol (10 ml/kg.d), olive oil (2 ml/kg.d) and pyrazole (25 mg/kg.d) for nine weeks [13]. The control group was intragastrically administrated with physiological saline. At 2, 4, 6 and 9 weeks, six rats of each group were sacrificed after fasting for 18 h. The samples were used for histopathology, plasma membrane purification and immunohistochemisty. In this study, mainly samples at 6 and 9 weeks were used for further studies Furthermore, in order to check if pyrazole and olive oil can induce liver fibrosis, 12 rats were intragastrically administrated with pyrazole (25 mg/kg.d) and olive oil (2 ml/kg.d) for 6 weeks or 9 weeks, and checked only through histopathology.

### Histopathology

Part of the liver from each rat was fixed in 4% paraformaldehyde in PBS and used for histopathology according to our previous work [12,14]. Liver tissues were stained by James and Masson stain [12,14]. Fibrosis score was evaluated according to the following standards: score 0, normal (no visible fibrosis); score 1, fibrosis present (collagen fiber present that extends from portal triad or central vein to peripheral region); score 2, mild fibrosis (mild collagen fiber present with extension without compartment formation); score 3, moderate fibrosis (moderate collagen fiber present with some pseudo lobe formation); and score 4, severe fibrosis (severe collagen fiber present with thickening of the partial compartments and frequent pseudo lobe formation). The rats with fibrosis score of 4 and ascites were diagnosed as liver cirrhosis.

### Preparation of rat liver PMs

PMs were purified through sucrose density gradient centrifugation twice according to the procedure described in our previous papers [14,15]. Briefly, the crude PM (CPM) at the top of 42.3% sucrose was collected and washed. The CPM pellets were transferred to SW-32 tubes, mixed with 50% sucrose, adding homogenization buffer or 50% sucrose to the mixture until the concentration of sucrose was 44%. Then sucrose step gradients containing 42.8%, 42.3%, 41.8%, 41.0%, 39.0%, and 37.0% sucrose were layered on the top. The purified PM (named PM) at the top of 37.0% sucrose was collected after centrifugation at 100,000 g for 6 h, washed with 40 mM HEPES with 1 mM PMSF twice, then stored at −80°C until further use.

### Two-dimensional electrophoresis (2DE) and gel staining

2DE was performed on an IPGphor isoelectronic focusing system (GE Healthcare, USA) and Bio-Rad Protein II electrophoresis apparatus according to our published papers [14,16]. For first-dimensional electrophoresis, 18 cm pH3-10 NL IEF strips from GE Healthcare were used. 1000 μg of protein from each sample was loaded and focused according to the following conditions: 30 V for 12 h, 500 V for 1 h, 1000 V for 1 h, 8000 V gradient for 30 min and 8000 V for 6 h up to 52.1 KVh. For second-dimensional electrophoresis, 11.5% separation gels were used and run in Bio-Rad Protein II electrophoresis apparatus. After completion of the second-dimensional electrophoresis, the gels were stained with G-250 Coomassie Brilliant Blue.

### Image acquisition and data analysis

The 2-DE gels were scanned by Imagescaner (GE Healthcare, USA) in transmission mode, and the image analysis was conducted with ImageMaster 2D software (GE Healthcare, USA). To get the comparable data for quantitative analysis, several key parameters in the image analysis were fixed as the constants. The volume of each individual protein spot was normalized by dividing the total volume of the entire image. The relative volume of each spot was used as an index to eliminate the density differences caused by the individual experimental errors. The difference in protein expression between liver fibrosis groups and the controls was estimated by two-sample *t*-test (p < 0.05) [17]. The threshold was defined as the significant change in spot volume was at least 2-fold upon the comparison of the average gels between the liver fibrosis and the controls.

### Identification of differentially expressed proteins by mass spectrometry

The differentially expressed proteins were analyzed by ESI-MS-MS mass spectrometry (esquire HCT, Bruker-Daltonics, Bremen, Germany) according to our recently published papers [14,16]. Briefly, the protein spots from Coomassie Blue-stained gels were destained with 50% ACN and digested with sequencing-grade modified trypsin 20 ng/ml over night at 37°C. The peptide extraction with 50% acetonitrile and 0.1% trifuoroacetic acid was freeze dried. The dried peptides were diluted with 2% ACN and 0.1% TFA in water and separated by Ultimate 3000 instruments (Dionex, Sunnyvale, CA, USA) through a C-18 reversed-phase nanocolumn (75 μm id × 15 cm length, 3 μm, PepMap™ (Dionex) after desalted by a C18 μ-precolumn (300 μm id × 5mm, 5 μm, PepMap™) (Dionex). The eluted peptides from the reversed-phase nanocolumn were on line injected to a PicoTip emitter nanospray needle (New Objective, Woburn, MA, USA) for real-time ionization and peptide detection by an ESI mass spectrometer. The MS conditions were as follows: capillary voltage, 1000–1500 V; dry gas (nitrogen) flow = 4.0 L/min; dry gas temperature = 150°C. For database search, search parameters were set as follows: enzyme, trypsin; allowance for up to one missed cleavage peptide; mass tolerance, 1.2 Da for MS and MS/MS mass tolerance, 0.6 Da; fixed modification parameter, carbamoylmethylation (C); variable modification parameters, oxidation (at Met); auto hits allowed (only significant hits were reported); results format as peptide summary report. Proteins were identified on the basis of Mascot searching scores > 33 and significance threshold of each peptide p < 0.05. The proteins identified by more than 4 peptides were accepted without manual check. For proteins identified by less than 4 peptides, each peptide was manually inspected to make sure a least one peptide with three or more continue y-or b-series ions (e.g., y4, y5, y6).

### Bioinformatics

The theoretical isoelectric point (pI) and molecular weight (MW) were extracted through the Mascot software. The subcellular location and function of the identified proteins were elucidated by UniProt knowledgebase (Swiss-Prot/TrEMBL) and Gene Ontology Database.

### Western blotting (WB)

50 μg of protein extracts were separated by electrophoresis in SDS-11.5% polyacrylamide gel and transferred to PVDF membrane (Millipore). Blots were incubated overnight at 4°C with the primary antibody (Primary antibodies used were as follows: ANXA3 Antibody (dilution: 1:1000,ProteinTech Group Inc, Chicago, USA), ANXA6 Antibody (dilution: 1:1500, ProteinTech Group Inc, Chicago, USA), mouse anti-Na^+^/K^+^-ATPase (1:5,000; Abcam, Cambridge, UK) and rabbit anti-prohibitin (dilution: 1:100; Abcam). After three washes with TBS-Tween, blots were incubated for 1 h at 20°C with secondary antibodies. Lastly the immune complexes were revealed by enhanced chemiluminescence and detected by exposure and development of X-ray film. In order to check the protein loading, the PVDF membrane after WB detection was stained by Coomassie Brilliant Blue R-250 according to previously reported [18] due to no suitable internal control in this study.

### Immunohistochemistry and semi-quantification of differentially expressed proteins

Immunohistochemistry was performed as described by previously reported methods [19,20]. Sections (5 μm) of paraffin-embedded tissues were deparaffinized, hydrated, and washed three times in PBS. Subsequently, the slides were incubated overnight at 4°C with anti-ANXA3 in a humidified chamber (dilution 1:100). The slides were washed in PBS for 3 times, incubated with Horseradish Peroxidase-conjugated antibody, and signals detected using a liquid 3, 3’-diaminobenzidine (DAB) staining kit (Gene Tech), counterstained with Hematoxylin-exon, dehydrated, mounted in Permount (Fisher Scientific) and imaged digitally by light microscopy using an Olympus BX40 equipped with a logenE PAS9000.

To semi-quantitatively analyze the expression of ANXA3 in liver tissue, each slide was randomly imaged for 10 times in 400-fold magnification. Cells with positive membrane expression of ANXA3 were counted in each image. The ratio of the average number of positive cells from rat liver tissues treated with ethanol for 6 and 9 weeks (total of 90 images every group) to that of controls (total of 90 images) was considered as the change rate of ANXA3 expression.

## Results

### Histopathological findings

**Figure 1:**
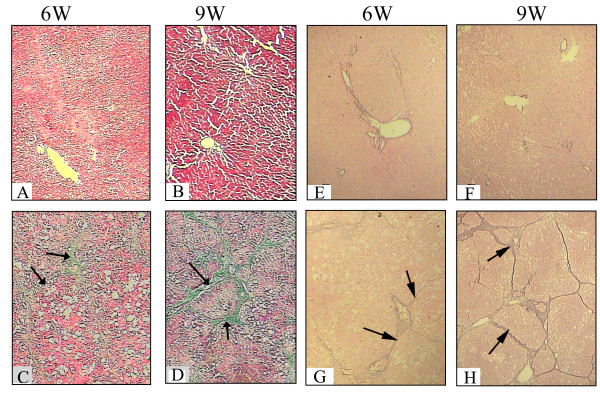
**Histopathological analysis of liver tissue stained by James and Masson staining.** Livers from normal (**A**, **B**, **E** and **F**) and alcoholic liver fibrosis (ALF) rats (**C**, **D**, **G** and **H**) at 6 and 9 weeks stained by Masson (A, B, C and D) or James staining (E, F, G and H). No substantial difference was found during the growth progress from 6 to 9 weeks in normal rat livers. For ALF models, the degree of liver fibrosis was increased gradually from S2 at 6 W, to S4 at 9 W as highlighted by arrowhead and showed in green or deep brown color.

As shown in Figure 1, the liver fibrosis model was successfully established. After treated with ethanol for 6 weeks, hepatic fibrosis with thin and separated small-sized pseudolobules (highlighted by arrowhead) was developed (Figure 1C and 1G), and diagnosed as S2. At 9 weeks, the collagen fiber became very thick and obvious pseudo lobes (highlighted by arrowhead) developed (Figure 1D and 1H), with the character of S4. However for the control group, no histopathological changes were detected from 6 to 9 weeks (Figure 1A, 1B, 1E and 1F).

### Purification of plasma membrane

**Figure 2:**
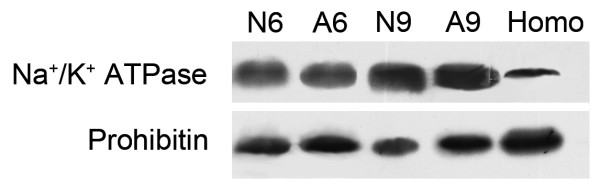
**Verification of PM purification through Western blotting.** 50 micrograms of homogenization and PM proteins were separated in 11.5% SDS-PAGE and then transferred to a PVDF membrane. The blots were probed with antibodies against organelle-specific proteins: anti-Na^+^/K^+^-ATPase for PM; anti-prohibitin for mitochondria. A9, N9 represent liver plasma membrane proteins from 9-weeks alcohol treated rat models and the control group. Homo means homogenization proteins from the liver of normal rat at 9 weeks.

PMs were obtained by two sequential sucrose gradient ultracentrifugations and their purity was evaluated by Western blotting (Figure 2). Fractions containing PM were identified by a PM marker enzyme, Na^+^/K^+^ ATPase and by decreased amounts of a mitochondrial marker, Prohibitin. According to the area of signal bands analyzed by Image J software (http://rsb.info.nih.gov/ij), PM were enriched for 2.6-, 2.4- fold at 6 week, and 3.6 and 3.8-fold at 9 week in the samples from normal and alcohol-treated respectively, in comparison with the original homogenate. While in the same samples mitochondrial markers were decreased for 0.7 and 0.8-fold and 0.5 and 0.7-fold respectively. The enrichment was basically consistent across different samples.

### 2DE profiles of PM and identification of differentially expressed proteins

**Figure 3:**
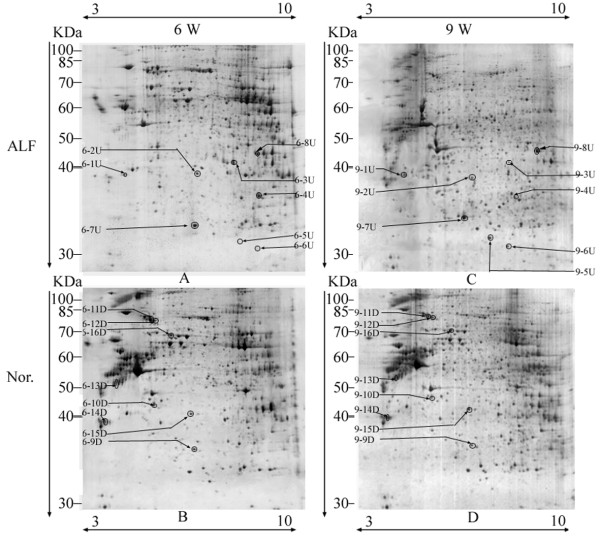
**Protein expression profiles of the ALF and the controls.** ALF represents alcohol induced liver fibrosis; Nor. means normal control. 6 W and 9 W represent the PM at 6 and 9 weeks. The up-regulated protein spots are labeled in the gels of ALF (**A** and **C**), the down-regulated protein spots are marked in the gels of normal (**B** and **D**). The molecular markers are shown in the left of each gel. The pI information is shown in the top of A and C, and the bottom of B and D.

**Figure 4:**
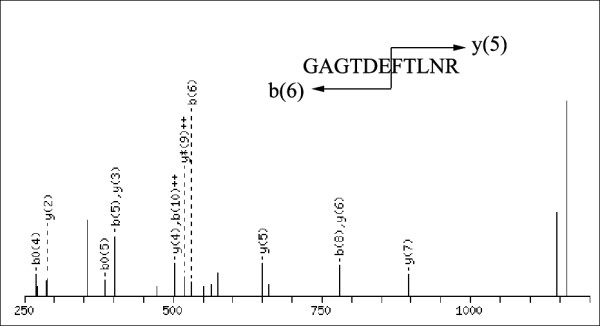
A representative MS/MS spectrum of peptide (GAGTDEFTLNR) from annexin A3 identified in this work.

To obtain a dynamic comparison of the difference in protein expression profile, the PM at 6 and 9 weeks were extracted and identified by LC-MS/MS. Through MS/MS analysis, 8 proteins with simultaneous up-regulation at 6 and 9 weeks were identified and marked with U in Figure 3A and 3C (ratio_ALF/Nor._ ≥ 2, *p* ≤ 0.05), while, 8 simultaneously down-regulated protein spots (ratio _ALF/Nor._ ≤ 0.5, *p* ≤ 0.05) were shown in Figure 3B and 3D, and marked with D. As shown in Table 1 and (Additional file 1: Table S1), 15 of 16 differentially expressed protein spots were identified by two or more peptides, only one identified by a single peptide. A represent MS/MS spectrum was shown in Figure 4. For the protein identified by single peptide, the MS/MS profile was checked manually and the MS/MS spectrum was shown in Additional file 2: Figure S1.

**Table 1:** Differentially expressed proteins identified in this work

***Spot***	***Accession NO.***	***Protein description***	***Sco.***	***pI***	***MW (KDa)***	***Cov.***	***Exp.6***	***Exp.9***	*** Function***	Cellular location*
6-1U,9-1U	ANXA5_RAT	Annexin A5	191	4.93	35.8	23%	2.36	2.4	binding	cytoplasm
6-2U,9-2U	THTM_RAT	3-mercaptopyruvate sulfurtransferase	194	5.88	33.2	29%	4.42	2.3	enzyme	Cytoplasm
6-3U,9-3U	CATL1_RAT	Cathepsin L1 precursor	83	6.37	38.2	26%	5.45	+∞	enzyme,binding	PM
6-4U,9-4U	ETFB_RAT	Electron transfer flavoprotein subunit beta	293	7.6	27.9	48%	3.77	+∞	enzyme	Mito.
6-5U,9-5U	MYH9_RAT	Myosin-9	168	5.49	227.6	2%	2.01	2.27	enzyme,binding	Cytoplasm
6-6U,9-6U	ARGI1_RAT	Arginase-1	101	6.76	35.1	15%	+∞	2.03	binding	PM
6-7U,9-7U	MYH9_RAT	Myosin-9	168	5.49	227.6	2%	2.00	5.69	binding,enzyme	Cytoplasm
6-8U,9-8U	ANXA2_RAT	Annexin A2	313	7.55	38.9	27%	3.69	2.92	binding	PM
6-9D,9-9D	PRDX1_RAT	Peroxiredoxin-1	49	8.27	22.3	9%	0.36	0.52	binding, peroxiredoxin, protein homodimerization	Cytoplasm, Melanosome
6-10D,9-10D	GBB2_RAT	Guanine nucleotide-binding protein G(I)/G(S)/G(T) subunit beta-2	39	5.6	38	3%	0.62	0.64	signal transduction, calcium channel regulator	PM
6-11D,9-11D	ANXA6_RAT	Annexin A6	466	5.39	76.1	45%	0.44	0.18	binding	PM, melanosome
6-12D,9-12D	ANXA6_RAT	Annexin A6	466	5.39	76.1	45%	0.50	0.17	binding	PM, melanosome
6-13D,9-13D	K2C8_RAT	Keratin, type II cytoskeletal 8	320	5.83	54	35%	0.16	0.56	binding, structural	PM
6-14D,9-14D	K1C18_RAT	Keratin, type I cytoskeletal 18	724	5.17	47.7	47%	0.31	0.65	structural	PM
6-15D,9-15D	ANXA3_RAT	Annexin A3	86	5.96	36.6	20%	0.64	0.27	binding,phospholipase A2 inhibitor	PM
6-16D,9-16D	PDIA3_RAT	Protein disulfide-isomerase A3 precursor	88	5.88	57	15%	0.65	-∞	enzyme	ER

*Sco* score, *MW* molecular weight, *Cov*. Sequence coverage, *Exp*. Expression (ratio of experimental group to control group), *PM* plasma membrane including external side of plasma membrane, plasma membrane, intermediate filament, *Mito*. Mitochondrion, *ER*. endoplasmic reticulum.

### Western blotting

**Figure 5:**
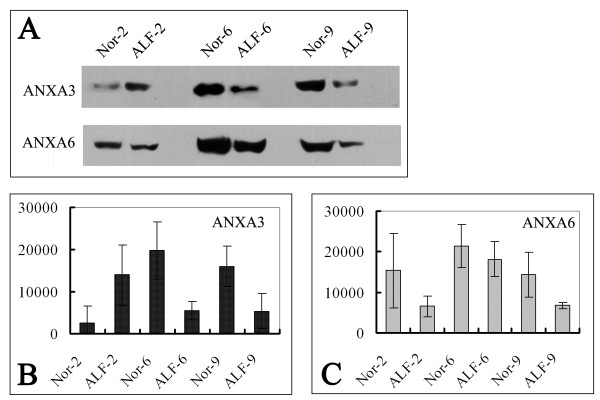
**Western blotting analyses of selected proteins.** 50 μg PM Protein extracts of 2, 6 and 9 weeks were separated by 11.5% separation gels and then transferred to a PVDF membrane. The blots were probed with antibodies against ANX3 and ANX6. **A**, The expression of ANX3 and ANX6 in 6 and 9 weeks rat model. ALF-2 and Nor-2 represent liver plasma membrane proteins from 2-weeks alcohol treated rat models and the control group. **B**, The signal intensity of specific bands of ANX3 analyzed by ImageJ software. **C**, The signal intensity of specific bands of ANX6. The data were presented as average value (from a protein loading of 50 ug) and standard deviation (n = 3).

The dynamic changes of two differentially expressed proteins: ANXA3 and ANXA6 were validated by western blot analyses in the PM from rat model. As shown in Figure 5, ANXA3 and ANXA6 were verified to be significantly decreased at 6 and 9 weeks in the liver PM of alcohol-treated rats compared with the controls, which was consistent with the results from 2DE. Furthermore, the same PVDF membranes stained with Coomassie Brilliant Blue R-250 showed that the protein loadings were constant in these samples (Shown in Additional file 3: Figure S2).

### Immunohistochemistry

**Figure 6:**
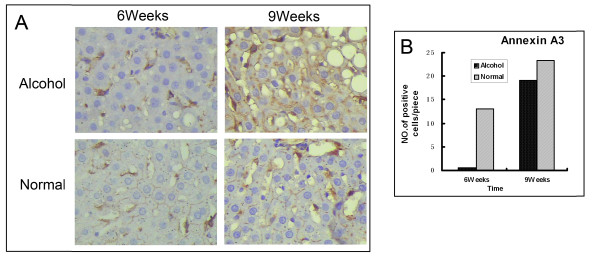
**Immunohistochemistry analyzing the expression of annexin A3. A**. Tissue immunostaining of select proteins identified in PM. Sections of rat liver tissue stained with antibodies recognizing the indicated proteins (×400). **B**. Semi-quantification analysis of alcohol-treated rats liver PM samples and control in 6 and 9 weeks.

Immunohistochemical studies were performed in the biopsy of rat model at 6 and 9 weeks using ANXA3 antibodies. The immunohistochemical analysis revealed a basically consistent result between the antibody staining and the proteome expression profiles obtained by 2-DE. At 6 weeks, very little positive signal was detected in the plasma membrane of rat liver hepatocytes in ethanol-treated group (Figure 6A), while strong signal was detected in the control. About 24-fold decrease was detected in ethanol-treated group compared with its controls through cell counting. To 9 weeks, a little difference was found between ethanol-treated group and its controls, with only 0.8-fold difference (Figure 6B).

## Discussion

Excessive consumption of alcohol contributes to alcoholic liver disease that is often silent until complications develop; therefore clinicians need a high index of suspicion to detect individuals with heavy alcohol consumption and evolving liver disease. In this work, a liver fibrosis rat model was successfully established by ethanol, olive oil and pyrazole treatment. Pyrazole is the inhibitor of alcohol dehydrogenase (ADH) [21]. Because rat has higher ethanol metabolic rate and greater tolerance to ethanol that differ from man [22] and the addition of pyrazole can slowdown the rate of ethanol metabolism in rat liver and increases in free radical production and cellular damage through peroxidation. Olive oil is added into the ALD model establishment process to raise the fat intake of model rats which can overload the liver cell fat metabolism, increase the production of lipid peroxidation, thereby increasing the degree of alcoholic liver injury. Furthermor, in order to make sure that pyrazole and olive oil only have help function in the progression of cirrhosis and do not induce liver fibrosis, a rat model treated with olive oil and pyrazole was developed and found that the liver was normal even treated for 9 week (Additional file 4: Figure S3). Due to the aim of this work to study proteins related to liver fibrosis, the control treated with physiological saline not with pyrazole and olive oil was used.

Generally, human liver tissues are more relevant and desirable to find index or biomarkers for alcohol liver disease. However, it is very difficult to study proteome related to alcohol liver disease using human tissue for the following reasons: (1) only tissues with very serious liver damage can be obtained; (2) It is very difficult to obtain enough sample for PM proteomic studies; and (3) greater individual variance in humans. Animal models of alcohol liver fibrosis not only provide a molecular insight into ethanol-induced cellular and protein dysfunction, but also afford a viable means to evaluate the usefulness of therapeutic agents aimed at prevention of disease progression. In this work, a rat model was built, in which mice were given ethanol over the course of 9 weeks [12]. Liver tissues at 6 and 9 weeks were detected to have fibrosis with S2 and S4 respectively.

So far, a lot of studies were reported about the proteins related to alcohol liver disease [23-26]. Some potential biomarkers were identified such as up-regulated C-reactive protein (CRP) [23] and PEDF [24] in serum from rats or humans with alcohol over-consumption. Alcohol was found to change the protein expression in mitochondrion [25] and induce global hepatic protein hyperacetylation in cytosolic and membrane proteins [27]. However, little was reported about the molecular basis of alcohol to liver injury in plasma membrane. In this work, double sucrose density gradient ultracentrifugation was used to purify rat liver plasma membrane. Through a 2DE-MS strategy combined with an optimized proteins extraction method [28], 8 of 14 differentially expressed proteins were PM or PM-related proteins. Although there were still 6 proteins from other subcellular organelles, including annexin A5, 3-mercaptopyruvate sulfurtransferase and Peroxiredoxin-1, this might be due to two reasons: 1) the multiple locations of proteins [29,30]. Mann et al. [30] have pointed out that 41% of all organelle proteins are found in more than one location; 2) the contamination of other organelles such as mitochondrial membrane, cytosol and endoplasmic reticulum. There is no technology that can remove other organelle proteins from the plasma membrane fraction completely so far. So our method can offer some help in studying lower abundant membrane proteins although plasma membrane is not enriched very well in this work.

According to GO database, classes of proteins affected by ethanol are involved in binding (57.5%), enzyme (31.5%) and cell structure activities (11.0%). These results indicated that the cell signal transport, biochemistry reaction and cell structure were mainly regulated during liver fibrogenesis after alcohol treatment.

According to the function and subcellular location of identified differentially expressed proteins as described above, we focused on the 5 proteins located in PM or were PM-related. They were ANXA2, ANXA6, K2C8, K1C18 and ANXA3. Of which, ANXA2, K2C8 and K1C18 were verified by western blot in our previous work [12]. In this work, ANXA3 and ANXA6 were verified by WB and ANXA3 was further verified by immunohistochemistry. ANXA3 was down-regulated in liver tissue at 6 and 9 weeks (Figures 5 and 6). However, ANXA3 was found to be up-regulated at 2 weeks [12]. This might due to the dynamic change of proteins in response to ethanol. According to the previous work described by Naama that proteins (even house keeping protein-GAPDH) can be dynamically regulated in the presence of drugs [31]. Annexin A3 is the inhibitor of phospholipase A2, also possesses anti-coagulant properties (from UniProtKB). Harashima, M’s work revealed that annexin A3 increases and plays important roles in the signalling cascade in hepatocyte growth in cultured hepatocytes [32]. Jung EJ’s proteomic analyses revealed that annexin A3 was significantly decreased in papillary thyroid carcinoma at both the protein and mRNA levels, compared with normal thyroid tissue [33]. Furthermore, several works revealed that the expression of annexin A3 was related to ovarian cancer. Yin J reported that sera from ovarian cancer patients contained significantly higher levels of annexin A3 compared with those from normal donors [34]. Yan X’s work revealed that increased expression of annexin A3 was a mechanism of platinum resistance in ovarian cancer [35]. There previous findings along with our data suggest that, annexin A3 has very important functions in disease development and treatment, which might be a potential drug target for treating alcohol-induced liver cirrhosis.

There were certain limitations of this work. After treated with ethanol for 6 and 9 weeks, hepatic fibrosis with stage of S2 and S4 were developed, but as indicated by Figure 1, the cellular components vary between stages of disease. In this work, whole liver was used rather than a purified cell type (e.g. hepatocyte). The differential protein expression might be due to difference in expression or simply changes in cell populations during the disease process. So protein expression verification in a purified cell type should be done in our future work. Furthermore, the functions of differentially expressed proteins need to be studied in the future.

## Conclusions

In summary, to the best of our knowledge, this is the first time to study the affect of alcohol on the liver cirrhosis through plasma membrane proteomics analysis. In this work, five novel proteins associated with alcohol induced hepatic fibrosis were detected. We have specifically identified annexin A3 and annexin A6 as a potential biomarker for predicting alcohol-induced liver cirrhosis. Further studies performed on clinical samples with alcohol-induced liver cirrhosis will be helpful to confirm the data obtained using this rat model in order to identify biochemical markers for diagnosis and prognosis of alcohol induced fibrosis, targets for therapy and treatment of alcohol induced fibrosis. Our findings validate the utility of proteomics in identifying novel biomarkers of disease pathogenesis and stage.

## Abbreviations

ALC: Alcoholic liver cirrhosis; 2-DE: Two-dimensional gel electrophoresis; PM: Plasma membrane; LC-MS: Liquid chromatography-mass spectrometry; HCT: High Capacity ion Trap; ANXA3: Annexin A3; WB: Western blotting; HE: Hematoxylin and Eosin; LC-MS/MS: Liquid chromatography combined with tandem mass spectrometry.

## Competing interests

The authors declare that they have no competing interests.

## Authors' contributions

XJ carried out the immunohistochemistry experiment, the mass spectrometry identification of differential proteins and drafted the manuscript. LY paticipated in the western blotting experiment and Image analysis of 2DE gels. Yanling Feng carried out the histopathology analysis. XP carried out the two-dimensional electrophoresis and gel staining. FM paticipated in the animal treatment. YY paticipated in the preparation of rat liver PMs experiment. XL paticipated in the preparation of rat liver PMs experiment and carried out the bioinformatics analysis. ZZ participated in experiment design. ZY participated in experiment design, directed part of experiments and revised the manuscript. LZ initials conceived of the study, experiment design and revised the manuscript. All authors read and approved the final manuscript.

## Supplementary Material

Additional file 1 **Table S1.** The peptide information of differentially expressed proteins identified by MS. Click here for file

Additional file 2 **Figure S1.** MS/MS fragmentation spectrum of TFVSGACDASIK from Guanine nucleotide-binding protein G(I)/G(S)/G(T) subunit beta-2 (accession number: GBB2_RAT). Click here for file

Additional file 3 **Figure S2.** Coomassie Brilliant Blue R-250 staining of PVDF membranes used as loading control for detection of ANXA3 and ANXA6 by Western blot analysis. Click here for file

Additional file 4 **Figure S3.** Histopathological analysis of liver tissue from rats treated with pyrazole and olive oil for 6 or 9 weeks. The slices were stained by Masson and HE staining. HE, Hematoxylin and Eosin staining; Mas, Masson staining. Click here for file
