# Supplementary figures and images for: A dynamic plasma membrane proteome analysis of alcohol-induced liver cirrhosis
Source: Proteome Sci. 2012 Jun 8;10:39. doi: 10.1186/1477-5956-10-39 (PMC3558348; doi:10.1186/1477-5956-10-39)

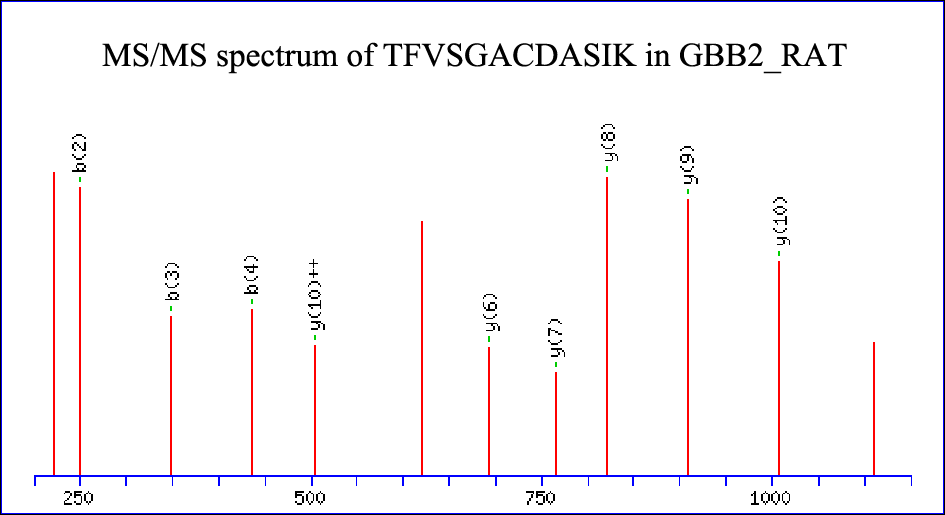

Supplement: Additional file 2 — Figure S1. MS/MS fragmentation spectrum of TFVSGACDASIK from Guanine nucleotide-binding protein G(I)/G(S)/G(T) subunit beta-2 (accession number: GBB2_RAT). [file 1477-5956-10-39-S2.tiff]

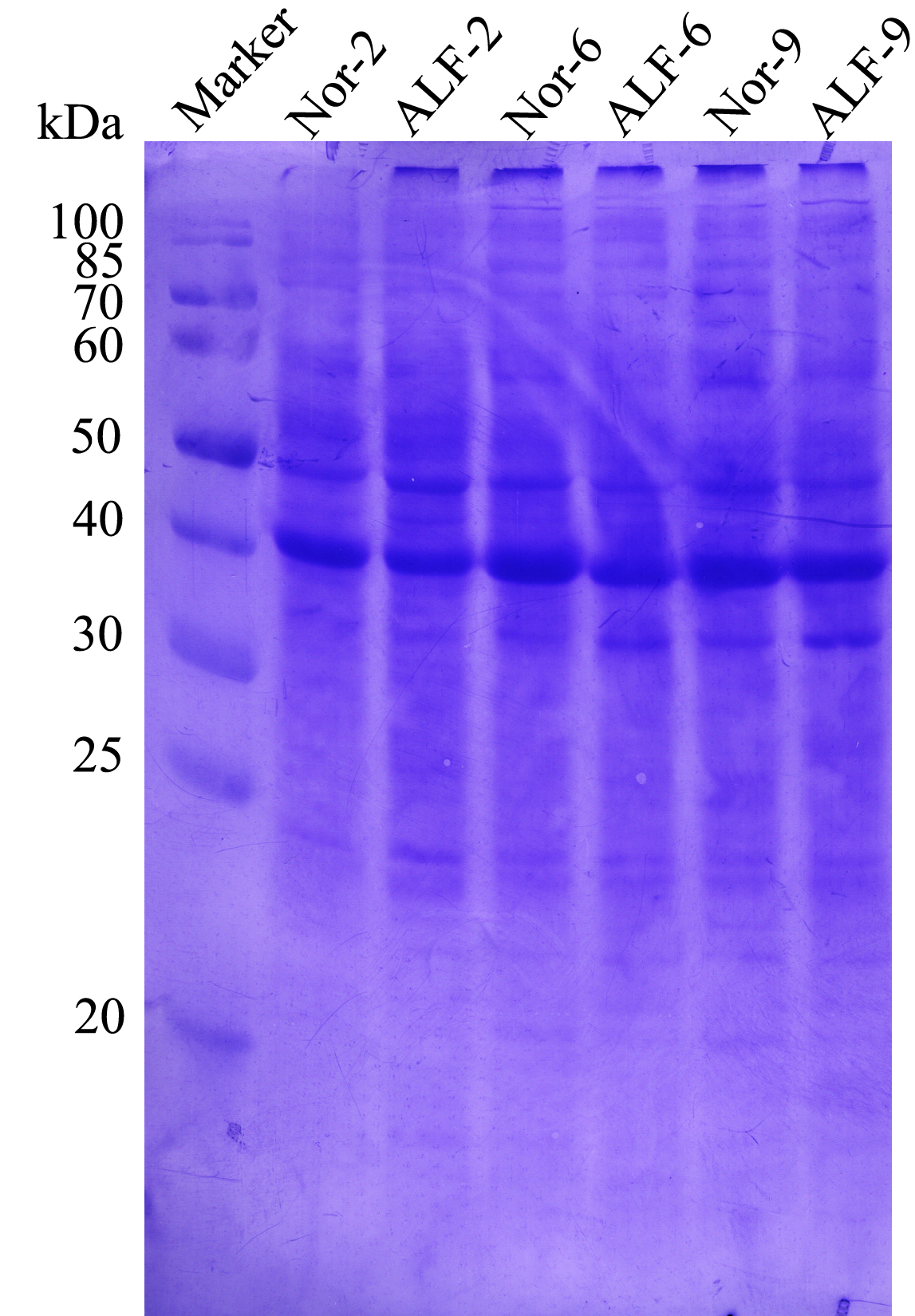

Supplement: Additional file 3 — Figure S2. Coomassie Brilliant Blue R-250 staining of PVDF membranes used as loading control for detection of ANXA3 and ANXA6 by Western blot analysis. [file 1477-5956-10-39-S3.jpeg]

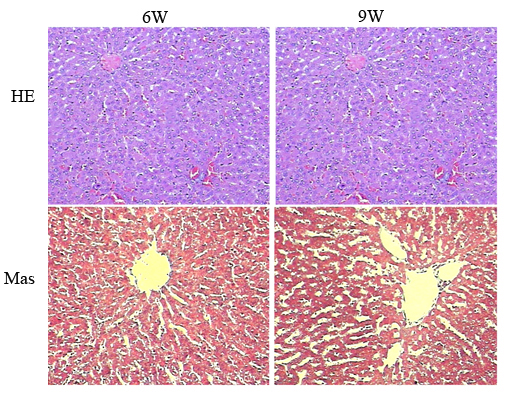

Supplement: Additional file 4 — Figure S3. Histopathological analysis of liver tissue from rats treated with pyrazole and olive oil for 6 or 9 weeks. The slices were stained by Masson and HE staining. HE, Hematoxylin and Eosin staining; Mas, Masson staining. [file 1477-5956-10-39-S4.jpeg]
